# Everybody's Page

**Published:** 1889-05-25

**Authors:** 


### Everybody's Page.

Goose-grease, when mixed with cocoa-butter, forms an admirable basis for ointment.

Among the North American Indians the hair grows to a remarkable length. One Crow chief is said to have had hair 10ft. 7in. long. He wore it rolled up with a leather strap, which made the *coiffure* weigh several pounds; but on occasions of ceremony he let it hang over him like a mantle.

Olives are now largely cultivated in California. There are several orchards of 15,000 trees, and more are planted every year. As it takes ten years for the trees to come to maturity, it is usual to plant grape-vines between them. The crop is gathered in December and January, and keeps very well.

Artificial honey, which is more common in the market than consumers know, is made of potato-starch and oil of vitriol. Some rash optimists think that they are sure of getting the genuine product of bees and flowers by purchasing honey in the comb. Deluded mortals! The exquisite white comb that pleases them is often made of paraffin wax.

Prosecutions for the adulteration of milk are not often successful, and they sometimes punish unjustly. The retail milkman may not be responsible for inferiority in the quality of the milk he sells; its poverty and lack of nourishing power may be caused by some quite temporary condition affecting the health of the cow.

In the first years of child-life, a bath night and morning s very desirable; but the morning bath, which is meant as a tonic and stimulant, should be cooler than the evening one, which should have a sedative action. Of course, it is the evening bath that is the more important for cleansing purposes, after the various forms of industry which children call play.

Onion juice is useful for gumming paper to metal. The cheaper kind of clock dials used to be printed on paper and then glued to a zinc foundation; but after a very short time paper and metal were apt to part company. Now the zinc is dipped into a strong solution of washing soda, and afterwards washed over with onion juice. If the paper is then pasted on to this, it is almost impossible to separate it from the plate.

He felt the patient's pulse, he sounded his chest, and took his temperature; then he said, "There's no help for it; you must go abroad for the winter." "But it costs so much; I can't afford it," answered the patient. The doctor considered for a minute, and then said, "Well perhaps, if you stay at home, and I visit you every day, I may pull you round." Whereat the patient said with strange promptitude, "Thank you, doctor; I hadn't thought of that. I'll go abroad.

We think that Dr. Hahn, of Berlin, has gone beyond the limits of reasonable experiment. A few months ago a woman was admitted into hospital for advanced recurrent cancer on the left side of the body. The case was evidently hopeless, but we do not think Dr. Hahn was justified in transplanting three grafts of cancerous skin to the right side of the body in order to prove that cancer could be caused by inoculation. The result of the experiment justified his hypothesis, for when the patient died, three months after, a microscopic examination distinctly showed the presence of cancer in the tissues under the transplanted skin; but we think that Dr. Hahn has distinctly infringed the tacit understanding on which a patient accepts hospital treatment. We cannot sacrifice humanity to pathology.

Wooden vessels may be rendered impervious to ordinary liquids by brushing them over with paraffin as long as the wood will absorb any.

"I'm not afraid of death, doctor," said the patient; "but it would be terrible to be buried alive." "Don't worry yourself about that," replied the doctor, cheerily; "I'll take good care it doesn't happen."

Pharmacy is receiving the attention of the Russian Government, and a plan is being prepared which will require that every chemist shall spend eight terms at a university, and will give a Master in Pharmacy an education and status equal to that of a Doctor of Medicine. The regulations and privileges will belong to both sexes equally.

Bather's cramp is often caused by the shock of cold water under certain conditions. Robust, middle-aged men seem more liable to it than others, though in any case it seems as if a constitutional predisposition were necessary. Great warmth of the body, fatigue, and sudden or severe muscular effort often tend to bring on an attack. Those who know themselves to be subject to cramp should not go into deep water when bathing.

The average baby ought to be thankful that its father is not an inventor. Mr. Edison has been guilty of what we should term great cruelty to his infant daughter. When she crows with delight, when she screams, every sound that comes from her lips is registered on the phonograph. He means to treasure the record till she grows up, and then let loose her infant utterances. He seems to think it will amuse her. We doubt it.

"Toddy" is a vernacular modification of the old Sanscrit word *tâli*, which denotes palm-wine. It became corrupted into *tâdi* by the vulgar, and finally came to signify other intoxicants. Probably the word was imported from India with other things in the good old days, when "John Company" set the pagoda-tree shaking; but it is not strange that some authorities derive it from the family name "Todd," and accredit some unknown member of the clan with being the inventor of the beverage "toddy."

One often reads pathetic stories of pet birds that die simultaneously with, or shortly after, their child owners. It sounds pretty, but the simple prose of the matter often is that the owners infected the birds. Canaries and other songsters will catch scarlet fever, measles, diphtheria, or almost any other human disease, and if left in the sick-room they are almost sure to be infected. Pet cats and small dogs, too, are often sacrificed in the same way, and in their case there is also the risk that they will go out and become the unwitting instruments of disseminating disease.

There has recently been a discussion on the origin of tetanus before the Paris Academy of Medicine. M. Verneuil has brought many arguments to bear on his theory that it is an infectious disease, capable of being transmitted from the domestic animals, especially horses, to man, and is of opinion that it originated with the equine race, and affects man by the ordinary methods of contagion. Dr. J. Pelletan, on the other hand, points out that tetanus appears usually as a result of injuries, with which, in most cases, horses have nothing to do, and also mentions the fact that in the islands of Oceania, where there are no horses, and never have been any, tetanus is by no means uncommon. The true origin of tetanus is still a mystery, and will probably remain so till the pathology of the nervous system is better known than it is at present.

